# Loss of Toll-Like Receptor 4 Function Partially Protects against Peripheral and Cardiac Glucose Metabolic Derangements During a Long-Term High-Fat Diet

**DOI:** 10.1371/journal.pone.0142077

**Published:** 2015-11-05

**Authors:** Ellen E. Jackson, Elisabeth Rendina-Ruedy, Brenda J. Smith, Veronique A. Lacombe

**Affiliations:** 1 Department of Physiological Sciences, Oklahoma State University, Stillwater, Oklahoma, United States of America; 2 Department of Nutritional Sciences, Oklahoma State University, Stillwater, Oklahoma, United States of America; 3 Harold Hamm Diabetes Center, University of Oklahoma Health Sciences Center, Oklahoma City, Oklahoma, United States of America; College of Tropical Agriculture and Human Resources , University of Hawaii, UNITED STATES

## Abstract

Diabetes is a chronic inflammatory disease that carries a high risk of cardiovascular disease. However, the pathophysiological link between these disorders is not well known. We hypothesize that TLR4 signaling mediates high fat diet (HFD)-induced peripheral and cardiac glucose metabolic derangements. Mice with a loss-of-function mutation in TLR4 (C3H/HeJ) and age-matched control (C57BL/6) mice were fed either a high-fat diet or normal diet for 16 weeks. Glucose tolerance and plasma insulin were measured. Protein expression of glucose transporters (GLUT), AKT (phosphorylated and total), and proinflammatory cytokines (IL-6, TNF-α and SOCS-3) were quantified in the heart using Western Blotting. Both groups fed a long-term HFD had increased body weight, blood glucose and insulin levels, as well as impaired glucose tolerance compared to mice fed a normal diet. TLR4-mutant mice were partially protected against long-term HFD-induced insulin resistance. In control mice, feeding a HFD decreased cardiac crude membrane GLUT4 protein content, which was partially rescued in TLR4-mutant mice. TLR4-mutant mice fed a HFD also had increased expression of GLUT8, a novel isoform, compared to mice fed a normal diet. GLUT8 content was positively correlated with SOCS-3 and IL-6 expression in the heart. No significant differences in cytokine expression were observed between groups, suggesting a lack of inflammation in the heart following a HFD. Loss of TLR4 function partially restored a healthy metabolic phenotype, suggesting that TLR4 signaling is a key mechanism in HFD-induced peripheral and cardiac insulin resistance. Our data further suggest that TLR4 exerts its detrimental metabolic effects in the myocardium through a cytokine-independent pathway.

## Introduction

Approximately, 2.1 billion people (nearly 30% of the global population) are overweight or obese [1]. In addition, the incidence of diabetes is anticipated to increase to epidemic levels in both industrial and developing countries over the next 2 decades [2]. Indeed, the incidence of diabetes has increased from 157 million people in 1980 to 347 million people in 2011 [2], and the CDC predicts that 1.7 million adults will be newly diagnosed with diabetes each year in the United States alone [3]. Insulin resistance, the hallmark of type 2 diabetes, is caused by a defect of insulin action resulting in dysfunctional glucose uptake into insulin-sensitive tissues (i.e., striated muscle and adipose tissue) such as the heart. At the molecular level, glucose uptake from the blood into the cell is the rate-limiting step of glucose utilization. Glucose transport is regulated by a family of specialized proteins called the glucose transport proteins (**GLUT**) [4]. GLUT4 is the major isoform in insulin-sensitive tissues, and as such is a key regulator of whole-body glucose homeostasis. Upon insulin stimulation, GLUT4 translocates from intracellular, cytoplasmic stores to the cell surface. Therefore, GLUT4 is responsible for insulin-stimulated glucose uptake [4, 5]. Although GLUT4 is the major isoform in the heart, recent evidence suggests that GLUT8, a novel isoform also highly expressed in the heart [6], could be a potential insulin sensitive GLUT [7].

As a result of these metabolic derangements, chronic diabetes mellitus can induce complications in multiple organs such as diabetic retinopathy, kidney failure, and heart disease [3]. Indeed, diabetes predisposes to cardiovascular disease, even in the absence of other risk factors [3]. However, the exact pathophysiological link between these disorders is unknown. It is well known that sub-clinical inflammation, defined as increased production and expression of pro-inflammatory cytokines, is associated with obesity [8] and diabetes [9–11]. Some cardiovascular diseases, especially diabetic cardiomyopathy, may have an inflammatory component in their pathogenesis [12, 13], although this potential pathogenic link has not been fully elucidated. The toll-like receptors (**TLR**) provide a possible link between diabetes, cardiovascular disease, and inflammation. TLRs are membrane receptors that play a key role in the innate and adaptive immune system [14]. While they are primarily expressed in immune cells, they have also been identified in insulin-sensitive tissues, including the heart [15, 16]. They recognize general pathogen-associated molecular patterns instead of specific molecular epitopes. Once a pattern binds, it triggers the release of pro-inflammatory cytokines (such as interleukin 6 [**IL-6**] and tumor necrosis factor alpha [**TNF-α**]), which in turn activate suppressor of cytokine signaling 3 (**SOCS-3**) [9]. Recently, TLR4 has emerged as a strong candidate for a cellular link between inflammation and insulin resistance. In addition to lipopolysaccharides from gram-negative bacteria [9, 14, 17], TLR4 can be activated by saturated free fatty acids during hyperlipidemic state associated with obesity and secondary to long-term ingestion of a high-fat diet (**HFD**) [18, 19]. In addition, TLR4 expression is primarily upregulated in visceral adipose tissue during insulin resistance [20]. Indeed, its activation has been implicated in the onset of insulin resistance in adipocytes of type 2 diabetic subjects [10]. Furthermore, humans with TLR4 mutations tend to be protected against developing diabetes [21]. However, to date, most studies have focused on immune cells or insulin-sensitive tissue other than the heart (i.e., skeletal muscle and adipose tissue) and the role of TLR4 in the heart is not well known [19, 22–24].

In the present study, we hypothesized that TLR4 signaling mediates both peripheral and cardiac insulin resistance. Therefore, our objectives were: 1) to determine whether mice that lack functional TLR4 will be protected against peripheral and cardiac derangements in glucose homeostasis during HFD-induced obesity and, 2) to identify the underlying intracellular signaling pathways. Our data suggested that activation of TLR4 signaling is detrimental to whole-body insulin responsiveness and glucose metabolism in the heart by altering the regulation of glucose transport through a cytokine-independent pathway.

## Materials and Methods

### Animal model

All procedures were approved by the Oklahoma State University Institutional Animal Care and Use Committee (#HE-092). Four-week-old male mice with a loss-of-function mutation in TLR4 (**TLR4-mutant,** C3H/HeJ) and age-matched wild-type control (C57BL/6N) mice were used in this study, as previously described [25]. C3H/HeJ mice have a naturally-occurring single point mutation in the cytoplasmic domain of TLR4 that makes the receptor non-functional. These TLR4-mutant mice have long been known to be resistant to lipopolysaccharide-induced septic shock [26, 27]. After a one-week acclimatization period, mice were randomly allocated to be fed either a normal diet (**ND**; 10% kcal from fat; AIN-93M) or a high-fat diet (**HFD**; 60% kcal from fat; Research Diets, Inc.; D12492) *ad libitum* for 16 weeks. Body weights and food intake were recorded weekly throughout the experimental period. At 16 weeks, an intraperitoneal glucose tolerance test was performed. Following a six-hour fast, 2.0 mg/kg glucose was administered IP and blood glucose concentrations were measured at time points from 0–120 min. Area under the curve (**AUC**) from the blood glucose response was calculated as a means to estimate insulin sensitivity. In addition, fasting plasma insulin was measured using a commercially available enzyme-based colorimetric insulin assay (Crystal Chem, Dowers Grove, IL). At 16 weeks, the mice were sacrificed using ketamine/xylazine and exsanguinated via the carotid artery. Whole hearts were collected and snap-frozen in liquid nitrogen for later analysis.

### Protein Extraction and Western Immunoblotting

Cardiac crude membrane protein extracts were obtained as previously described [20, 24, 28–31]. Briefly, 40-60mg of sample was homogenized in homogenization buffer (210mM sucrose, 40mM NaCl, 2mM EGTA, and 30mM HEPES) and lysed in lysis buffer (1.167M KCl and 58.3mM sodium pyrophosphate) before being centrifuged at an average speed of 227,220g (Rotor type 50.2Ti, Beckman-Coulter) for 90min at 4°C. The pellets were resuspended in a final buffer (1mM EDTA, 10mM Tris, 4% SDS) before being centrifuged at 3,000g for 45 minutes. The supernatant was collected and frozen at -80°C before use. Total protein lysates were collected by homogenizing tissue samples in total protein homogenization buffer (50mM Tris HCL, 150mM NaCl, 1% Triton, and 1:500 protease inhibitor cocktail) with a polytron. The samples were then centrifuged at 4°C and 800g. The supernatants were collected and frozen at -80°C before use.

Western blots were performed on crude membrane protein extracts (GLUTs) and total protein lysates (phosphorylated AKT at the Ser473 site [pAKT], total AKT, SOCS-3, IL-6, and TNF-α). Briefly, samples were run on a 12% SDS-PAGE gel at 120 volts for 120 min (GLUT4, GLUT8, pAKT, total AKT) or for 95 min (IL-6, SOCS-3, and TNF-α), as previously described [20, 24, 30, 31]. Proteins were transferred onto a PVDF membrane in buffer for 75min at 100V. Membranes were blocked at room temperature for 1hr in 5% milk or 2% goat serum in TPBS, then incubated overnight at 4°C with the primary antibody of choice (polyclonal rabbit anti-human GLUT4, AbDSerotec 4670–1704, 1:750; polyclonal rabbit anti-human GLUT8, Bioss bs-4241R, 1:500; monoclonal rabbit anti-human pAKT, Cell Signaling 4060, 1:1000; monoclonal rabbit anti-mouse total AKT, Cell Signaling 4061, 1:1000; polyclonal rabbit anti-human SOCS-3, Abcam 16030, 1:1000; rabbit anti-mouse IL-6, Santa Cruz 1265-R, 1:350; polyclonal rabbit anti-mouse TNF-α, Abcam 9739, 1:3333). The next day, membranes were incubated with horseradish peroxidase-linked secondary antibodies (for pAKT and panAKT, Cell Signaling 7074, 1:2000, polyclonal goat anti-rabbit; for all others, Thermo-Scientific PA1-903, varying concentrations, polyclonal donkey anti-rabbit). Quantitative determination of protein was performed by autoradiography after revealing the antibody-bound protein by enhanced chemiluminescence reaction (KPL). Equal protein loading was confirmed by reprobing each membrane for calsequestrin protein expression (Thermo-Scientific PA1-903, 1:2500, polyclonal, rabbit anti-dog). The optical density of each band was measured using GelPro Analyzer software (Media Cybernetics).

### Statistical Analyses

All data were analyzed by two-way ANOVA using Sigma Stat v. 3.5. If a treatment and/or genotype interaction was found, then the Student-Newman-Keuls method was performed for multiple comparisons. Correlations were analyzed by linear regression. Statistical significance was set at p<0.05. All data are presented as mean ± standard error.

## Results

### TLR4-mutant mice were partially protected against long-term HFD-induced peripheral glucose intolerance and hyperinsulinemia

**Fig 1:**
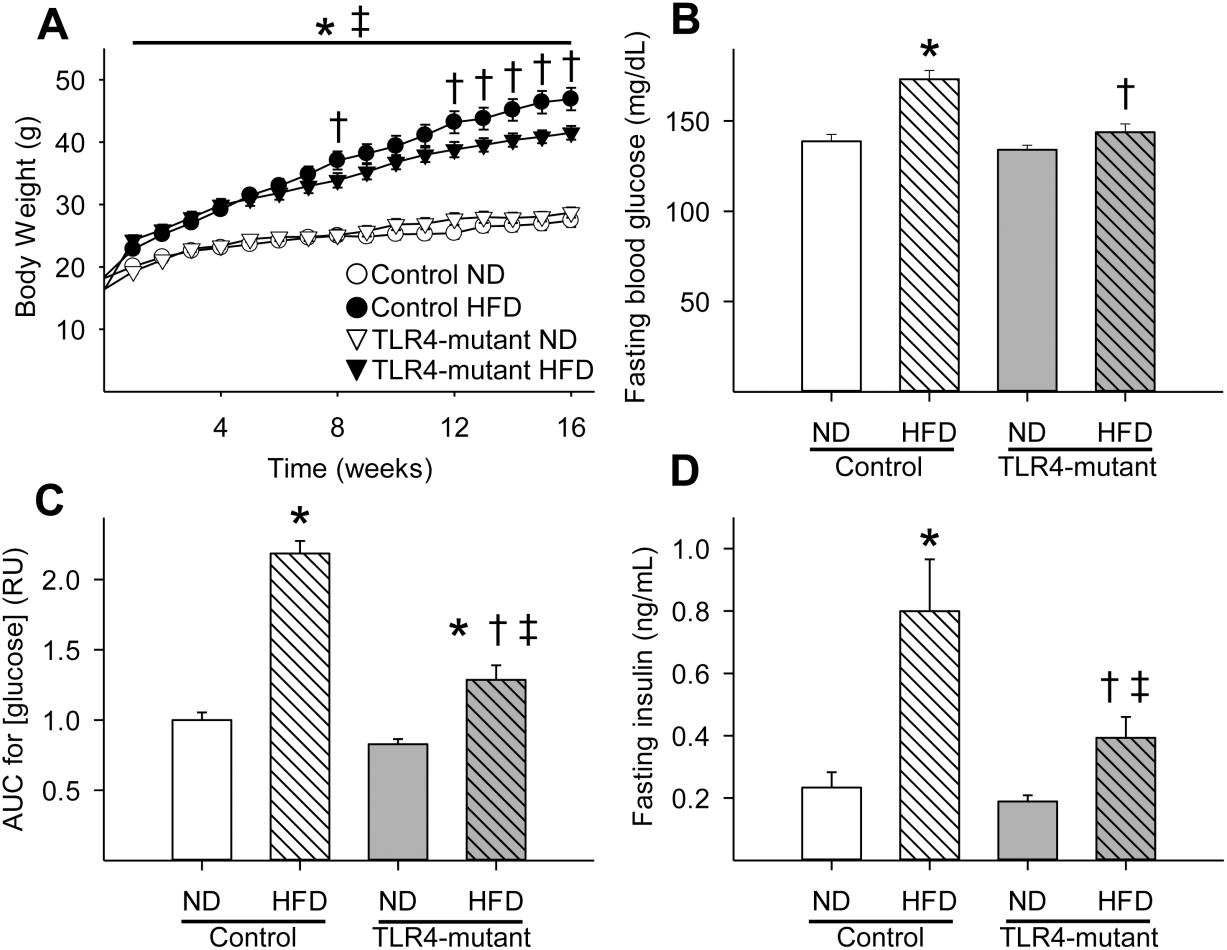
TLR4-mutant mice were partially protected against obesity (A), hyperglycemia (B), peripheral glucose intolerance (C), and hyperinsulinemia (D) induced by a HFD. AUC = area under the curve for [glucose] measured during IPGTT. HFD = high-fat diet; ND = normal diet; * p<0.05 vs. control fed a ND, † p<0.05 vs. control fed a HFD, ‡ p<0.05 vs. TLR4-mutant fed a ND; n = 8–21 per group.

By the end of the first week, mice fed a HFD weighed significantly more (p<0.05) than their counterparts fed a normal diet (Fig 1A). HFD-induced obesity persisted through the end of the study. Body weight of TLR4-mutant and control mice fed a normal diet did not differ significantly over the course of the study (Fig 1A). However, TLR4-mutant mice fed a HFD weighed significantly less than control mice fed a HFD starting at week 12 (Fig 1A). Similarly, the HFD-fed mice had increased body fat (as relative % of body weight and total mass, P<0.05) compared to mice fed a normal diet. In addition, TLR4-mutant mice had significantly decreased body fat (as relative % of body weight and total mass), without a change in total lean mass, as compared to control mice after 16 weeks on a HFD (S1 Fig). As expected, food intake was less (p<0.05) in mice fed a long-term HFD compared to mice on a normal diet. However, food intake was similar in TLR4-mutant mice compared to control mice fed a HFD (S2 Fig). In addition, after 16 weeks on a HFD, control mice developed mild hyperglycemia while TLR4-mutant mice remained euglycemic (Fig 1B). While all mice fed a HFD developed impaired glucose tolerance compared to mice fed a normal diet, the control group had significantly poorer glucose tolerance than TLR4-mutant mice after 16 weeks on a HFD (Fig 1C and S3 Fig). Furthermore, mice fed a HFD developed hyperinsulinemia compared to their counterparts fed a normal diet. However, TLR4-mutant mice fed a HFD had significantly lower plasma insulin concentrations than the control group fed a long-term HFD (Fig 1D).

### Role of TLR4 in the regulation of cardiac glucose transport

**Fig 2:**
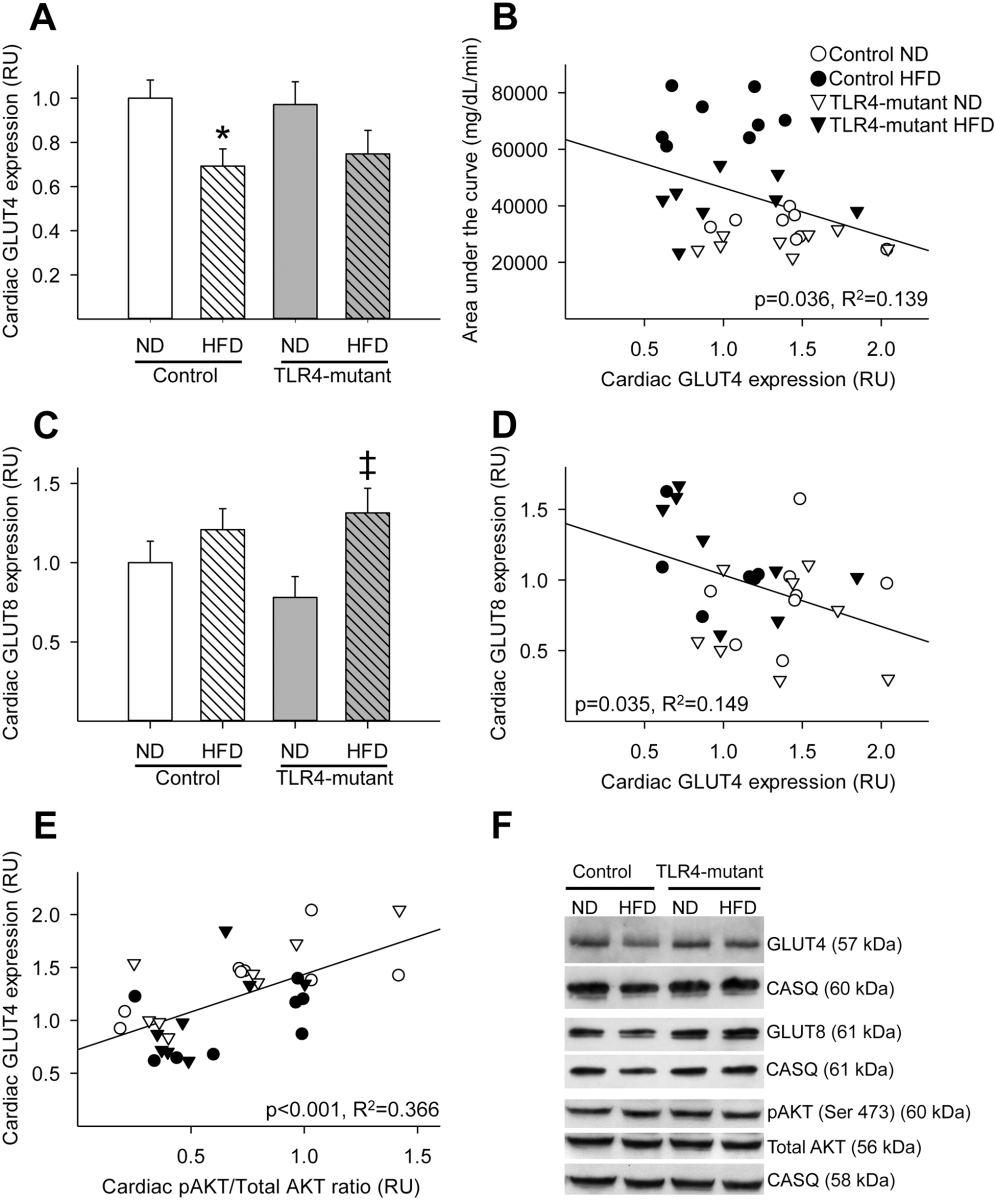
Downregulation of crude membrane GLUT4, but not GLUT8, protein content in the heart was attenuated in TLR4-mutant mice fed a HFD. (A) Mean ± SE of cardiac GLUT4 crude membrane protein content (relative to control ND). (B) Linear correlation between cardiac GLUT4 protein content and whole-body glucose tolerance, measured as AUC (R^2^ = 0.139, p = 0.036). (C) Mean ± SE of cardiac GLUT8 crude membrane protein content (relative to control ND). (D) Linear correlation between cardiac GLUT4 and GLUT8 expression (R^2^ = 0.149, p = 0.035). (E) Linear correlation between cardiac phosphorylated AKT (Ser473)/total AKT ratio and cardiac GLUT4 protein content (R^2^ = 0.366, p<0.001). (F) Representative Western blots of cardiac GLUT4, GLUT8, phosphorylated AKT (Ser473), total AKT and their corresponding calsequestrin used as a loading control. RU = relative units; AUC = area under the curve for [glucose] measured during IPGTT; HFD: high-fat diet; ND: normal diet. Trendlines show the line-of-best fit for all four groups taken together. * p<0.05 vs. control fed a ND, ‡ p<0.05 vs. TLR4-mutant fed a ND; n = 6–8 per group.

In order to investigate whether TLR4 signaling could modulate glucose homeostasis in the heart, we quantified GLUT4 protein content in cardiac crude membrane protein extracts by Western Blotting, a method previously validated to estimate GLUT translocation to the cell surface [30] (Fig 2). We reported that control mice fed a HFD had decreased cardiac crude membrane GLUT4 content compared to control mice fed a normal diet. In contrast, TLR4-mutant mice fed a HFD did not have a significant decrease in crude membrane GLUT4 content compared to TLR4-mutant mice fed a normal diet (Fig 2A). Additionally, cardiac GLUT4 protein expression negatively correlated with area under the curve obtained from the glucose tolerance test (Fig 2B), suggesting that cardiac glucose transport contributes to whole-body glucose homeostasis. Surprisingly, protein expression of GLUT8, a novel GLUT isoform, was increased in the myocardium in mice fed a HFD, with the increase being statistically significant in the TLR4-mutant mice fed a HFD (Fig 2C). As cardiac GLUT8 and GLUT4 protein expression followed opposite expression patterns, we investigated the potential relationship between these two GLUTs by performing linear regression. We found that GLUT4 and GLUT8 protein content were significantly negatively correlated in the myocardium of TLR4 mutant and control mice fed either a normal or long-term HFD (Fig 2D).

To further investigate the regulation of glucose transport in the myocardium, we quantified the expression of a key protein involved in the downstream insulin signaling pathway, namely AKT. Although no significant differences in total and phosphorylated AKT were observed between groups (Fig 2F), cardiac GLUT4 protein content positively correlated (p<0.05) with the pAKT (Ser307)/total AKT ratio (Fig 2E). In contrast, cardiac GLUT8 protein content did not significantly correlate with the pAKT (Ser307)/total AKT ratio (data not shown).

### Role of long-term HFD and TLR4 in the induction of inflammation in the myocardium

**Fig 3:**
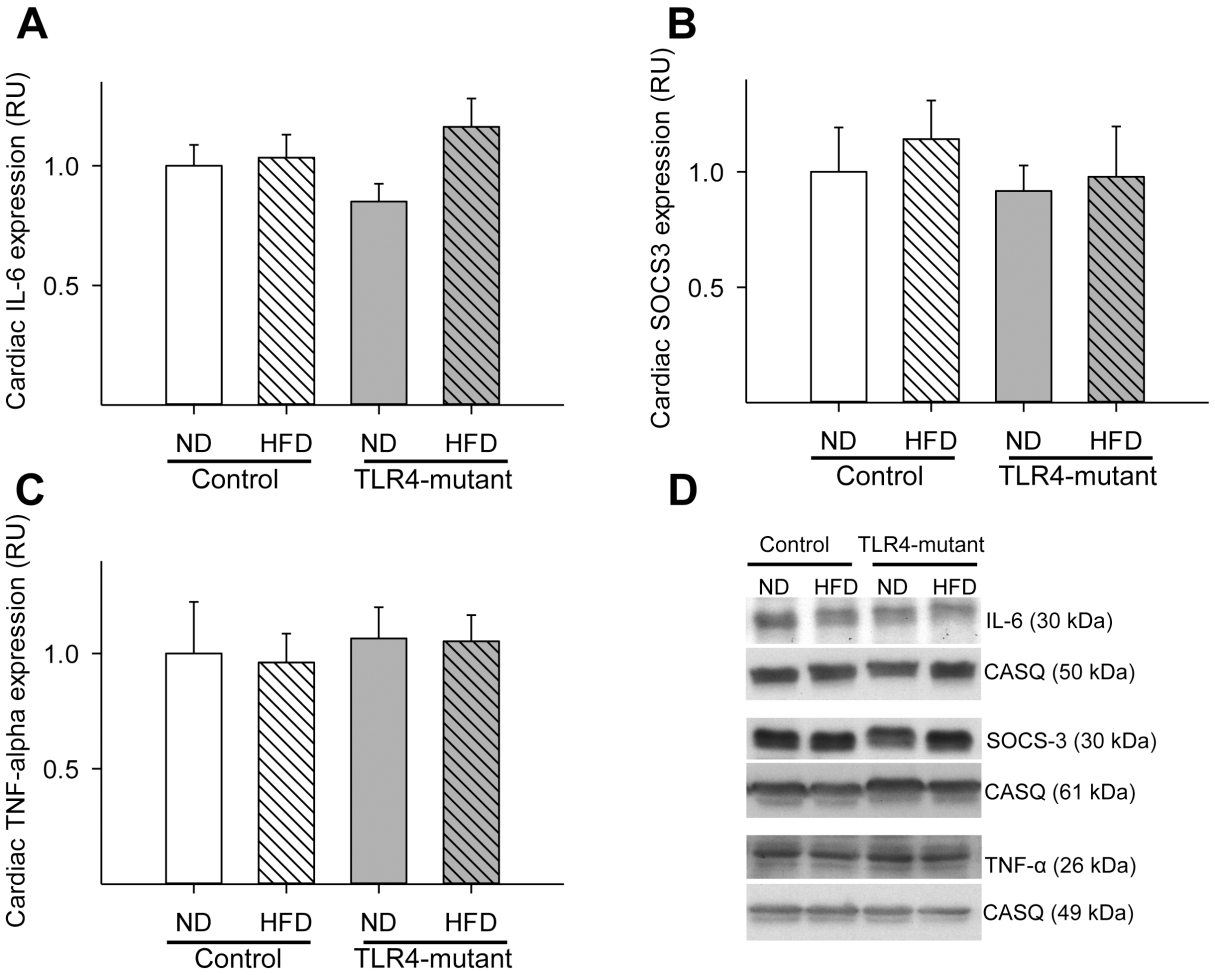
Lack of increased pro-inflammatory cytokine expression in the myocardium following a long-term high fat diet. (A) Mean ± SE of cardiac IL-6 protein content from cardiac total lysate. (B) Mean ± SE of cardiac SOCS-3 protein content from cardiac total lysate. (C) Mean ± SE of TNF-alpha protein content from cardiac total lysate. (D) Representative Western Blots of IL-6, SOCS-3, TNF-alpha and their corresponding calsequestrin (loading control). RU = relative units; HFD = high-fat diet; ND = normal diet. Values are relative to relative to control ND; n = 6–8 per group.

**Fig 4:**
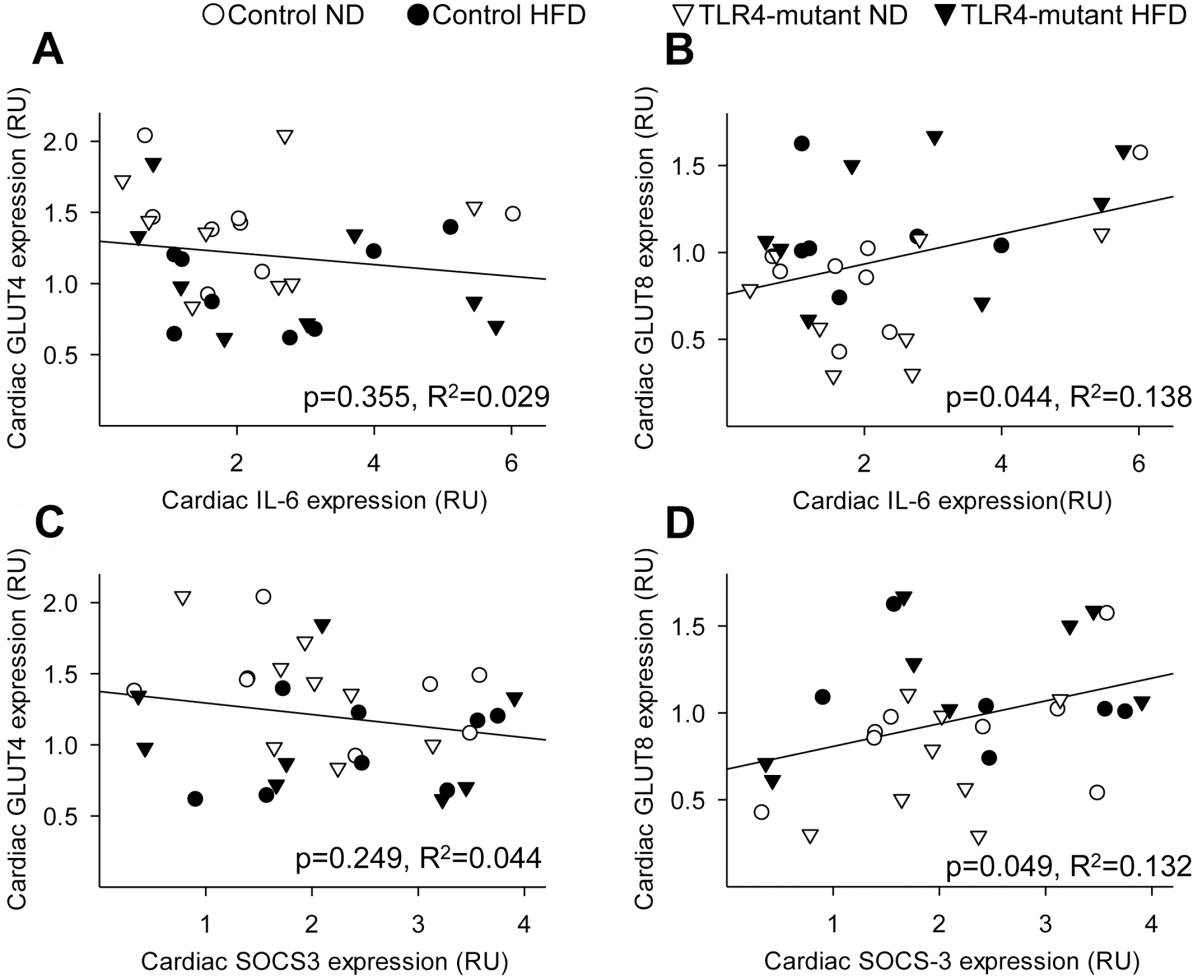
Correlations between pro-inflammatory cytokines and glucose transport in the heart. (A) Linear correlation between cardiac IL-6 protein expression and GLUT4 protein expression (R^2^ = 0.029, p = 0.355). (B) Linear correlation between cardiac IL-6 protein expression and GLUT8 protein expression (R^2^ = 0.138, p = 0.044). (C) Linear correlation between cardiac SOCS-3 protein expression and GLUT4 protein expression (R^2^ = 0.044, p = 0.249). (D) Linear correlation between cardiac SOCS-3 protein content and GLUT8 protein content (R^2^ = 0.132, p = 0.049). RU = relative units; HFD = high-fat diet; ND = normal diet. Trendlines show the line-of-best fit for all four groups taken together. n = 6–8 per group.

In order to investigate whether feeding a HFD for 16 weeks induces cardiac inflammation, protein expression of major pro-inflammatory cytokines was quantified as direct or indirect measure of TLR4 signaling (i.e., IL-6/TNF-α and SOCS-3, respectively). Surprisingly, no significant differences in SOCS-3, IL-6, or TNF-α expression were observed in the myocardium between groups (Fig 3). To investigate the potential interplay of glucose metabolism and inflammation in the heart, linear regression between the expression of pro-inflammatory cytokines and GLUTs were performed. Interestingly, cardiac GLUT8, but not GLUT4, protein content correlated positively (p<0.05) with both IL-6 and SOCS-3 expression (Fig 4). TNF-α protein content did not correlate with cardiac protein expression of either GLUT isoform (data not shown).

## Discussion

Using an integrative physiological approach, these data suggested a link between TLR4 signaling and peripheral and cardiac metabolic defects. At the organism level, the loss-of-function mutation in TLR4 partially protected against HFD-induced hyperinsulinemia and impaired glucose tolerance. At the molecular level, the TLR4 loss-of-function mutation partially rescued crude membrane GLUT4 protein expression in the heart in the face of whole-body insulin resistance, suggesting that TLR4 affects cardiac glucose metabolism as in other insulin-sensitive tissues, such as skeletal muscle [22] and adipose tissue [23]. Overall, these findings support the hypothesis that activation of TLR4 signaling is detrimental to whole-body glucose metabolism, including in the heart. Finally, these results suggest that HFD-induced cardiac glucose dysregulation precedes the development of cardiac inflammation and that insulin resistance is among the earliest pathophysiological changes in the myocardium.

In agreement with previous studies, feeding a HFD induced peripheral insulin resistance, as evident by obesity, hyperglycemia, hyperinsulinemia and glucose intolerance compared to mice fed a normal diet [22, 32, 33]. Additionally, TLR4-mutant mice were partially protected against these metabolic disorders, suggesting that activation of TLR4 contributes to HFD-induced peripheral insulin resistance [22, 34]. In addition, food intake was similar in the TLR4 mutant compared to control mice fed a long-term high fat diet, suggesting that the observed changes in body weight and glucose dysregulation are linked to the loss of TLR4 function rather than a change in energy intake.

Cardiac insulin resistance, characterized by impaired insulin stimulated glucose uptake into the myocardium, is concomitantly observed with peripheral insulin resistance in type 2 diabetic subjects [35]. Indeed, cardiac insulin resistance is an early pathogenic event, which occurs as little as one-and-a-half weeks during HFD-induced type 2 diabetes [36]. In our rodent model, we reported that feeding a high-fat diet for 16 weeks reduced crude membrane protein content of GLUT4 in the hearts of control mice, suggesting that long-term high-fat feeding and concomitant obesity induces cardiac insulin resistance. Although it has been shown that TLR4 mediates myocardial inflammation and ischemic injury [37], its role during cardiac metabolic derangements has not been well established. To our knowledge, only one study demonstrated that TLR4 deficiency delays cardiac lipid accumulation in a rodent type 1 diabetes model [16]. In the present study, downregulation of membrane-bound GLUT4 protein expression in the heart was attenuated in TLR4-mutant mice. These data suggest that TLR4-mutant mice are partially protected against HFD-induced cardiac insulin resistance and that TLR4 activation modulates myocardial glucose transport. Our data further suggest that GLUT4 contributes to whole-body glucose homeostasis, underscoring the importance to understand the regulation of glucose transport in the heart during physiological and pathophysiological conditions. Therefore, the present study provides novel insights into the interplay of glucose metabolism and immunity in the heart.

In order to sustain its high energy demand, the rate of glucose utilization in the heart is greater than in skeletal muscle and adipose tissue, despite the ability of the myocardium to use other substrates [38,39]. Therefore, it has been suggested that cardiac glucose transport plays a key role in regulating whole-body glucose homeostasis [39]. The present data supports this concept since crude membrane GLUT4 content was significantly correlated with insulin sensitivity (estimated by area under the glucose curve) in healthy and insulin resistant mice. Although the heart is a major organ that utilizes glucose, the downstream signaling pathways that modulate glucose transport are not well elucidated in the heart compared to other insulin-sensitive tissues. In skeletal muscle, activation of IRS-1 protein is followed by the activation of several kinases, which in turn recruit the pivotal serine/threonine protein kinase, namely, AKT. Phosphorylation of AKT at the Ser-473 site triggers GLUT4 translocation from intracellular stores (inactive site) to the cell surface (active site) [4]. We reported a positive correlation between AKT phosphorylation and GLUT4, supporting that AKT phosphorylation regulates GLUT4 translocation in the heart as in other insulin-sensitive tissues [9, 40].

Although GLUT4 is the major GLUT isoform in insulin-sensitive tissue, GLUT4 knockout mice do not develop hyperglycemia [41], suggesting that other GLUT isoforms may be involved in the regulation of whole-body glucose homeostasis. Recently, GLUT8, a class III member of the GLUT family, has emerged as a novel isoform regulating glucose transport in striated muscle [42] and adipose tissue [43]. Importantly, GLUT8 is one of the major GLUT transcripts expressed in the heart [6]. Although GLUT8 has been reported as an insulin-dependent isoform in blastocytes [7], its functional role during physiological and pathophysiological conditions in the myocardium is unknown. Our data highlights a new role of this novel isoform during a long-term HFD, and, to our knowledge, this is the first report of GLUT8 protein expression in the heart. Interestingly, in the current study, we reported that cardiac crude membrane protein content of GLUT8 was upregulated with a HFD. We further observed an inverse correlation between GLUT4 and GLUT8 protein content in the healthy and diabetic myocardium. Thus, one could speculate that GLUT8 may act as a compensatory mechanism for the observed HFD-induced downregulation of GLUT4 expression in the myocardium. Similarly, we previously demonstrated that active cell surface GLUT12 (also a member of the class III GLUT) was upregulated in the heart of type 1 diabetic subjects [39]. Therefore, class III GLUT isoforms represent an intriguing therapeutic avenue. We further reported a significant positive correlation between cardiac crude membrane protein content of GLUT8 and pro-inflammatory cytokines, namely IL-6 and SOCS-3, but not GLUT4, suggesting that GLUT8 may be modulated by proinflammatory cytokines. Interestingly, GLUT8 knockout mice fed a high-fructose diet showed improved glucose tolerance as compared to their wild-type and fructose-fed peers [44]. This observation prompted the authors of this study to speculate that GLUT8 may be a metabolically detrimental glucose transporter. Therefore, further studies are needed to determine the metabolic role of GLUT8 in diabetic patients.

One surprising result from this study is that there was no evidence of long-term HFD inducing cardiac inflammation, as demonstrated by the lack of increase in pro-inflammatory cytokine tissue expression between groups. This is contrast with previous studies that reported an increased expression of pro-inflammatory cytokines in skeletal muscle and adipose tissue [23] when the animal is challenged with a HFD. Since diet-associated inflammation is often subclinical [33, 45], one would expect that tissue markers of inflammation would be present. Thus, this data suggests that the heart may be less vulnerable to HFD-induced inflammation than other insulin-sensitive tissues. Indeed, we recently reported that prolonged supraphysiologic insulin infusion decreases TLR4 expression in the heart of mammalians without increasing production of pro-inflammatory cytokines, suggesting that hyperinsulinaemia exerts an anti-inflammatory effect on the heart [46]. Taken together, these studies indicate the relative insensitivity of striated muscle to inflammation as compared to adipose tissue [47]. In addition, these results suggest that HFD-induced cardiac glucose dysregulation precedes the development of cardiac inflammation. Indeed, growing evidence suggests that alteration in glucose metabolism and insulin resistance are among the earliest pathophysiological changes in the diabetic myocardium, preceding structural and functional changes [38].

Interestingly, pro-inflammatory cytokines such as TNF-α and IL-6, key players of the TLR4 signaling pathways, have also been implicated in the pathogenesis of impaired glucose uptake and insulin resistance in humans and rodents[11]. For instance, high-dose treatment of anti-inflammatory salicylate prevented lipid-induced insulin resistance in skeletal muscle [48]. In addition, suppressor of cytokine signaling (SOCS) proteins, which are feedback inhibitors of pathways induced by cytokines, may indirectly downregulate insulin signaling, resulting in reduced glucose transport into cells, the hallmark of insulin resistance [9]. However, the role of SOCS-3 as a modulator of glucose transport in the myocardium is unknown as most studies only report its upregulation in association with whole-body insulin resistance [20] or lipid infusion [49]. Therefore, we investigated whether activation of TLR4 signaling (through TNF-α, IL-6 and/or SOCS3) could modulate glucose metabolism in the heart. In the present study, we did not report any significant difference in protein expression of TNF-alpha, IL-6 and SOCS3 during HFD-induced cardiac insulin resistance. This is in contrast with studies in other insulin-sensitive tissues that reported a link between TLR4 activation and insulin resistance through the upregulation of pro-inflammatory cytokines [11, 50]. Therefore, our data suggest that TLR4 may exert its detrimental effects on glucose metabolism in the myocardium through a cytokine-independent pathway. Indeed, it has been suggested that activation of TLR4 signaling directly alters the phosphorylation status of IRS, which in turn impairs AKT activation and thus induces insulin resistance in adipose tissue [9]. However, a similar negative-feedback mechanism in the myocardium has not been reported. In the current study, we found no significant differences in expression of total or phosphorylated AKT expression (or its ratio) between TLR4-mutant and control groups, suggesting that TLR4 modulates glucose transport in the heart through an AKT independent pathway. Therefore, future studies are required to investigate the intracellular mechanisms involved in TLR4-induced alteration of cardiac glucose transport.

In summary, this study provides novel insight on the role of immunity during cardio-metabolic diseases. While a lack of functional TLR4 partially protects against cardiac glucose metabolism dysregulation induced by a long-term high fat diet, its mechanism of action appears to occur through an AKT- and cytokine-independent pathway. Finally, our data suggest that inactivation of aberrant TLR4 function offer a novel therapeutic strategy to prevent and/or treat obesity-induced cardiac insulin resistance and its associated cardiovascular co-morbidities.

## Supporting Information

S1 Fig TLR4-mutant mice had less fat body mass compared to control mice after 16 weeks on a HFD. A) Mean ± SE of fat and lean mass as percentage of total body weight (B) Mean ± SEM of fat mass. (C) Mean ± SEM of lean mass (n = 8 per group); HFD = high-fat diet; ND = normal diet; * p<0.05 vs. control fed a ND, † p<0.05 vs. control fed a HFD, ‡ p<0.05 vs. TLR4-mutant fed a ND.
(TIF)

S2 Fig Food intake was similar in TLR4-mutant mice vs. control mice fed a HFD. Data are mean ± SEM of daily food intake (n = 30 per group). HFD = high-fat diet; ND = normal diet; * p<0.05 vs. control fed a ND, ‡ p<0.05 vs. TLR4-mutant fed a ND.
(TIF)

S3 Fig Glucose tolerance test curves in mice after 16 weeks on HFD. Data are mean ± SEM (n = 8 per group). HFD = high-fat diet; ND = normal diet; *p<0.05 vs. control fed a ND, † p<0.05 vs. control fed a HFD, ‡ p<0.05 vs. TLR4-mutant fed a ND.
(TIF)
