# Supplementary figures and images for: Loss of Toll-Like Receptor 4 Function Partially Protects against Peripheral and Cardiac Glucose Metabolic Derangements During a Long-Term High-Fat Diet
Source: PLoS One. 2015 Nov 5;10(11):e0142077. doi: 10.1371/journal.pone.0142077 (PMC4634760; doi:10.1371/journal.pone.0142077)

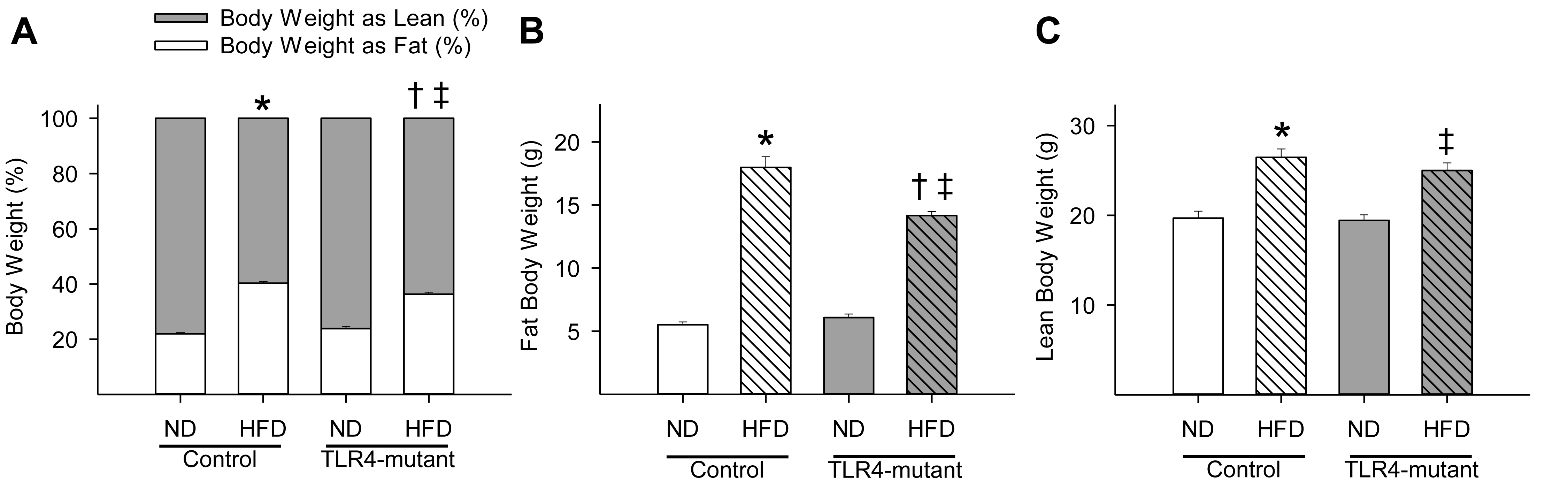

Supplement: S1 Fig — A) Mean ± SE of fat and lean mass as percentage of total body weight (B) Mean ± SEM of fat mass. (C) Mean ± SEM of lean mass (n = 8 per group); HFD = high-fat diet; ND = normal diet; * p<0.05 vs. control fed a ND, † p<0.05 vs. control fed a HFD, ‡ p<0.05 vs. TLR4-mutant fed a ND. (TIF) [file pone.0142077.s001.tif]

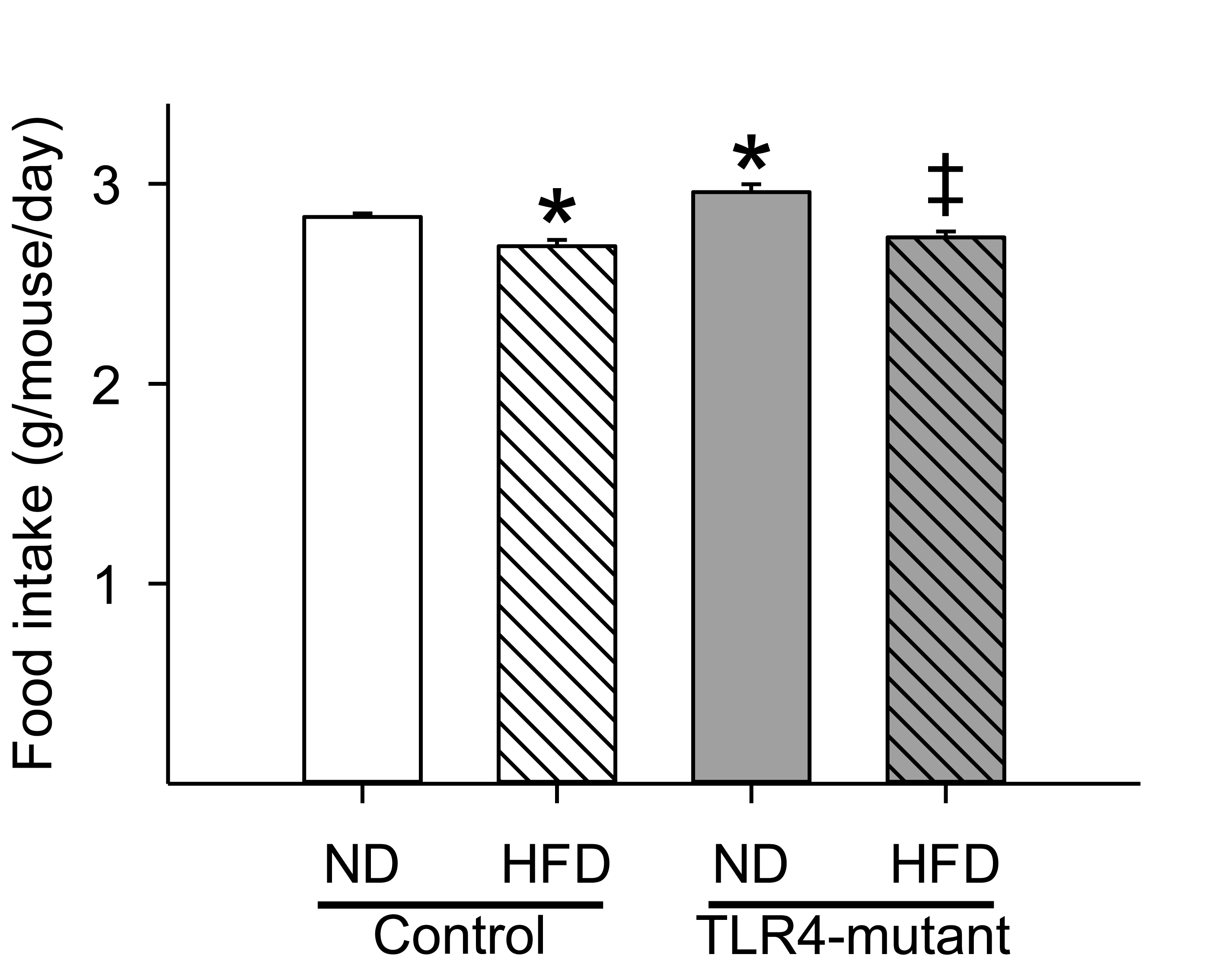

Supplement: S2 Fig — Data are mean ± SEM of daily food intake (n = 30 per group). HFD = high-fat diet; ND = normal diet; * p<0.05 vs. control fed a ND, ‡ p<0.05 vs. TLR4-mutant fed a ND. (TIF) [file pone.0142077.s002.tif]

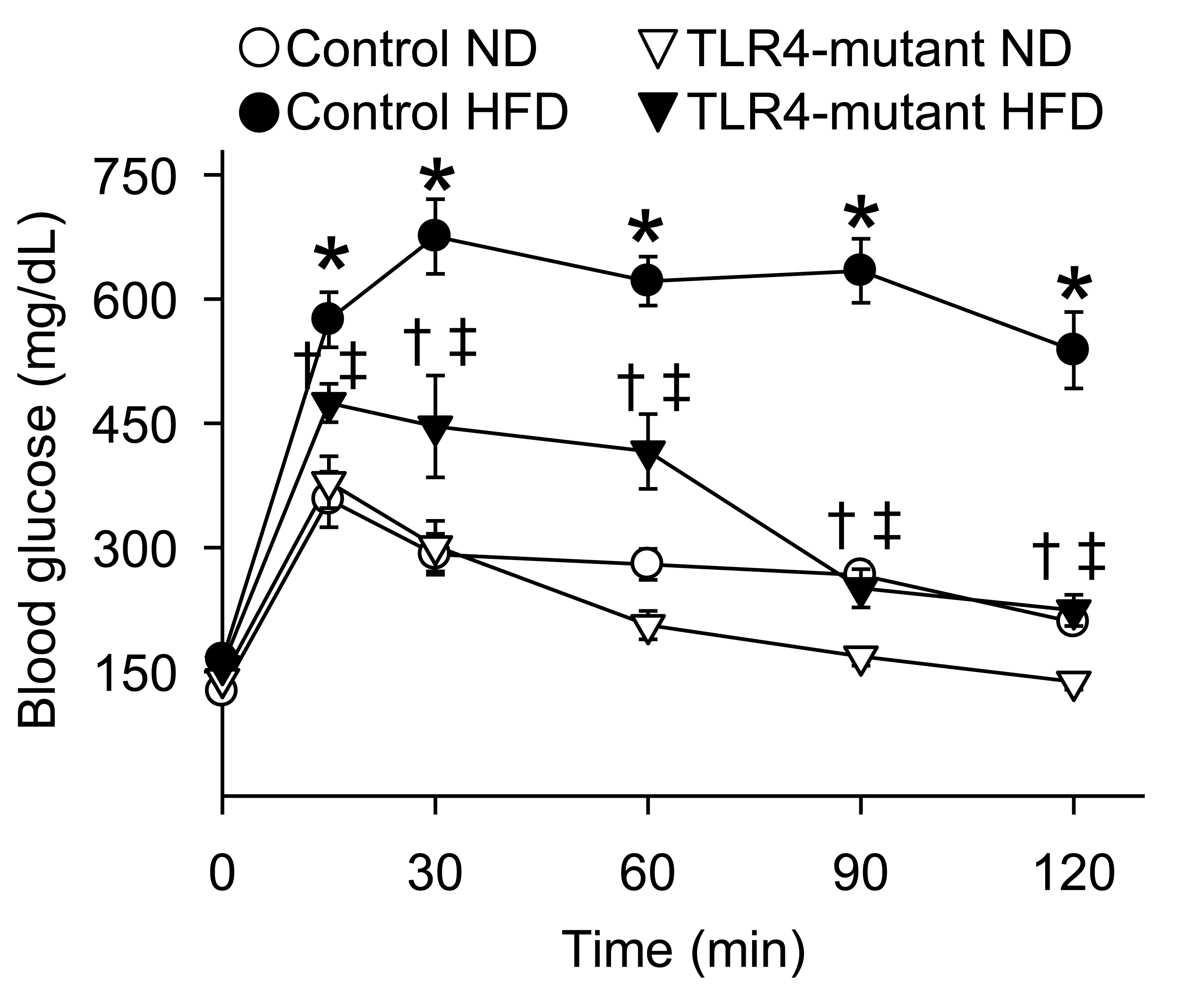

Supplement: S3 Fig — Data are mean ± SEM (n = 8 per group). HFD = high-fat diet; ND = normal diet; *p<0.05 vs. control fed a ND, † p<0.05 vs. control fed a HFD, ‡ p<0.05 vs. TLR4-mutant fed a ND. (TIF) [file pone.0142077.s003.tif]
